# Impact of a recolonizing, cross-border carnivore population on ungulate harvest in Scandinavia

**DOI:** 10.1038/s41598-020-78585-8

**Published:** 2020-12-10

**Authors:** Camilla Wikenros, Håkan Sand, Johan Månsson, Erling Maartmann, Ane Eriksen, Petter Wabakken, Barbara Zimmermann

**Affiliations:** 1 Grimsö Wildlife Research Station, Department of Ecology, Swedish University of Agricultural Sciences, 730 91 Riddarhyttan, Sweden; 2 Faculty of Applied Ecology, Agricultural Sciences and Biotechnology, Campus Evenstad, Inland Norway University of Applied Sciences, 2480 Koppang, Norway

**Keywords:** Biodiversity, Time series

## Abstract

Predation from large carnivores and human harvest are the two main mortality factors affecting the dynamics of many ungulate populations. We examined long-term moose (*Alces alces*) harvest data from two countries that share cross-border populations of wolves (*Canis lupus*) and their main prey moose. We tested how a spatial gradient of increasing wolf territory density affected moose harvest density and age and sex composition of the harvested animals (n = 549,310), along a latitudinal gradient during 1995–2017. In areas containing average-sized wolf territories, harvest density was on average 37% (Norway) and 51% (Sweden) lower than in areas without wolves. In Sweden, calves made up a higher proportion of the moose harvest than in Norway, and this proportion was reduced with increased wolf territory density, while it increased in Norway. The proportion of females in the adult harvest was more strongly reduced in Sweden than in Norway as a response to increased wolf territory density. Moose management in both countries performed actions aimed to increase productivity in the moose population, in order to compensate for the increased mortality caused by wolves. These management actions are empirical examples of an adaptive management in response to the return of large carnivores.

## Introduction

Predation from large carnivores and human harvest are two main mortality causes shaping population dynamics for many ungulate populations worldwide^1–4^. The return of large carnivores and ungulates in multi-use landscapes induce increased interactions between humans and wildlife^5,6^. These interactions can have implications for human land use and may require humans to adapt to a new situation, as well as wildlife to adapt to humans in shared landscapes, for example through shifting diet or alteration of habitat use or activity pattern^7–9^.

Large carnivores depredate on livestock and compete for game, mainly ungulates, with humans. Whereas depredation on livestock is mitigated by interventions such as carnivore-proof fencing^10^, the increased impact on ungulate mortality may require adjusted harvest strategies to avoid overexploitation and secure a sustainable yield^11^. For most ungulate species, human harvest has a larger impact on population growth compared to predation (per capita kill) as hunters generally select for adult animals at a higher rate than large carnivores^12,13^. Moreover, since reproduction is closely linked to the age and sex composition of the population^14^, harvest of certain categories can strongly affect population growth, and thus the possible harvest yield^12,15^. For example, harvest should mainly be directed towards calves if the aim is to maximize the number of harvested animals^16^. Harvest adjustments in response to the local establishment of large carnivores will therefore also have important consequences for ungulate population growth and possible harvest yield^12,17^.

In the absence of large carnivores, and with the introduction of stand forestry practices and hunting regulations throughout the twentieth century, the Scandinavian moose (*Alces alces*) population has shown a tremendous growth and been one of the most heavily harvested ungulate populations in the world^4^. In Scandinavia, moose hunting has long cultural traditions and constitutes great recreational as well as economic value^18,19^. The moose harvest is organized in management units with hunting teams managing the moose population within the same area year after year. This management system creates an incentive for the hunters to strive for a sustainable harvest on a long-term basis. A harvest over several years that is greater than the annual sustainable harvest will inevitably lead to reduced moose density and decreased harvest in the future. However, during the same time period, forestry has developed to be an important part of the national economy in Scandinavia^20^. An increase in the moose population size, resulting in locally high moose densities can also cause extensive browsing damage to young forest stands that in turn may lead to severe economic loss for forest owners^21,22^. This has created a national-wide system with multiple goals for the moose population. This includes the objective to keep browsing damage on commercially valuable tree species at an acceptable level by forest management^23^, while at the same time limit the moose population by harvesting at a level that results in a sustainable yield that is acceptable to hunters.

The recolonization of wolves (*Canis lupus*) in Scandinavia has further accentuated the challenge of combining hunter harvest and forestry, since the establishment of wolves could lead to a significant decrease in the moose harvest yield without a concomitant increase in moose density as wolf predation replaces human harvest^24^. However, an alternative to reducing total moose harvest may be to change the age and sex composition of harvested animals to compensate for the increase in mortality caused by wolf predation^11,24^. In a previous study during the early phase of wolf recolonization, Swedish hunters responded to the establishment of wolf territories by reducing their harvest and/or changing the composition of harvest structure towards a lower proportion of adult females^17^. The first years after wolf establishment, this reduction in harvest was actually greater than required to compensate for the effect of wolf predation^17^. This rapid response by hunters after wolf establishment contrasts with the more commonly observed time-lagged functional response between hunter harvest rates and changes in ungulate population densities^25^.

Several theoretical studies have evaluated various possible harvest strategies for moose and how harvest, for a given density, can be adjusted to reach different types of management goals such as maximizing the amount of meat, total number of harvested animals, or large males^11,24,26^. Wolves generally show a strong preference for moose calves year round^27,28^. Therefore, the proportion of calves in the population will be reduced as a direct consequence of predation by wolves. Thus, a lower ratio of calves in the harvest can therefore be expected.

In this study, we extended the approach used in previous studies, both in time and space to examine the effect of wolf recolonization on harvest of moose along a landscape gradient during 23 years of wolf population increase (1995–2017). We analysed harvest data of moose from two bordering countries (Norway and Sweden) which share joint cross-border populations of wolves and moose. More specifically, we investigated how territory density of wolves in a 2-year (hereafter short-term wolf index) and in a 5-year (hereafter long-term wolf index) time perspective affected harvest density and the age and sex composition of the harvested animals (proportion of calves and adult females in harvest). We predicted that the growth of the wolf population would result in (1) a large-scale reduction in moose harvest, and (2) a change in the age and sex composition of harvested moose with adult female moose harvested at a lower rate in areas with increasing wolf density. For the proportion of calves in harvest, we foresaw two alternative, diverging responses to increasing wolf territory density; either a decrease in calf harvest as a compensation for the wolves’ selection for calves, or an increase in calf harvest in order to spare animals with a higher reproductive value in the population.

## Results

### Harvest density of moose

**Figure 1:**
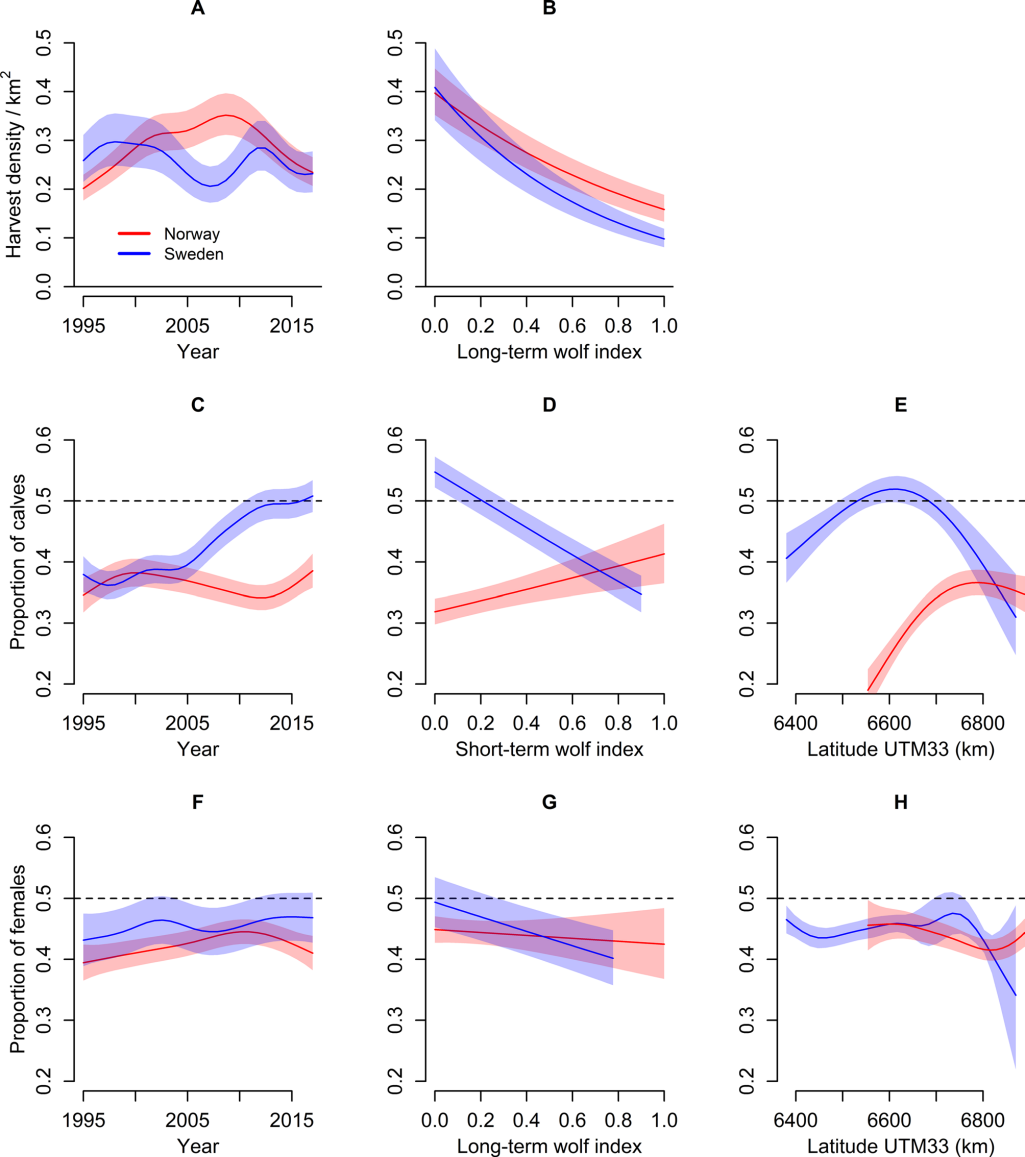
Predicted density (per km^2^) of harvested moose (**A**,**B**), proportion of calves in total harvest (**C**–**E**), and proportion of females in adult harvest (**F**–**H**) in Norwegian (red) and Swedish (blue) moose management units from 1995 until 2017 (**A**,**C**,**F**), average annual wolf territory density during the last 5 years (long-term wolf index) or 2 years (short-term wolf index) (**B**,**D**,**G**), and latitude (**E**,**H**). The predictions show average and 95% confidence intervals and are from generalized additive mixed models (GAMM) without the random factor (ID of management unit). Reference values were year 2012, wolf index 0.25, and latitude 6700 km. Dashed line represents an equal proportion of harvested calves and adults (**C**–**E**) and adult females and males (**F**–**H**).

We used harvest data from a total of 2228 annual moose management units (hereafter MMUs) during 1995 to 2017 (missing data for 233 MMU-years), including a total of 221,951 and 327,359 moose harvested in the study area in Norway and Sweden, respectively. Harvest density was at a comparable level in Norway and Sweden, but the temporal pattern differed between countries (Tables 1, 2, Fig. 1A). In Norway, harvest density increased from 1995 to 2010, and then declined to approximately the same level in 2017 as in 1995. In Sweden, there were two peaks in harvest density, one around 2000 and another in 2012, each followed by a decrease leading to the lowest harvest density in 2008 and 2017 (Fig. 1A). Both countries showed a reduction in harvest density for the last 6-year period. The long-term wolf index explained the relationship between wolf presence and total harvest density better than the short-term index (Table 1). The top model predicted that harvest density in MMUs with an average annual wolf territory density (long-term wolf index of 0.5) was 37% ± 5% (mean ± 2*SE*) and 51% ± 2% lower than the harvest density in MMUs without wolves, for Norway and Sweden, respectively (Fig. 1B). The harvest density did not vary with latitude (Table 2).

**Table 1:** Generalized additive mixed models (GAMM) assessing the effect of short- and long-term wolf index (Wolf_short_ or Wolf_long_) as linear functions, year (Year_s_) and latitude (Lat_s_) as smooth functions, and country (Norway, Sweden) on harvest density of moose (per km^2^), proportion of calves in total harvest, and proportion of females in adult harvest in Sweden and Norway during 1995–2017.

Response variable	Intercept	Wolf_short_	Wolf_long_	Country	Year_s_	Lat_s_	AICc weight	df	ΔAICc
Harvest density	X		X*C	X	X*C		0.41	108	0
	X		X*C	X	X*C	X*C	0.33	109	0.46
	X		X*C	X	X*C	X	0.26	109	0.95
	X		X		X*C		0	107	30.77
	X	X*C		X	X*C	X*C	0	108	288.80
	X				X*C		0	105	1440.86
	X				X*C	X	0	105	1441.22
	X				X*C	X*C	0	105	1441.46
	X						0	89	5653.38
	X			X			0	89	5653.92
Proportion of calves in total harvest	X	X*C		X	X*C	X*C	0.96	67	0
	X		X*C	X	X*C	X*C	0.04	67	6.36
	X		X*C	X	X*C		0	80	27.97
	X		X		X*C		0	87	132.27
	X				X*C	X*C	0	82	212.79
	X				X*C		0	86	221.15
	X				X*C	X	0	86	221.46
	X			X			0	65	1241.33
	X						0	73	1252.26
Proportion of females in adult harvest	X		X*C	X	X*C	X*C	0.92	33	0
	X	X*C		X	X*C	X*C	0.07	33	5.22
	X		X*C	X	X*C		0.01	36	8.89
	X		X		X*C		0	39	18.19
	X				X*C	X*C	0	31	20.86
	X				X*C	X	0	33	24.17
	X				X*C		0	38	33.82
	X			X			0	25	60.58
	X						0	30	67.18

X*C indicates that the specific variable was included in the model as an interaction with country. The management unit ID was included in all models as a smooth random variable. For each model, AICc weights, degrees of freedom (df), and difference in AICc relative to the highest-ranked model (ΔAICc) are shown.

**Table 2:** Summary of the best generalized additive mixed models (GAMM) used to explain harvest density of moose (per km^2^), proportion of calves in total harvest, and proportion of females in adult harvest in Sweden and Norway during 1995–2017.

Response variable	Explanatory variables	β	SE	z	p
Harvest density	Intercept	− 1.0110	0.0591	− 17.096	< 0.001
	Wolf_long_	− 0.919	0.0734	− 12.51	< 0.001
	Country	0.00394	0.108	0.036	0.971
	Wolf_long_ × Country	− 0.508	0.0839	− 6.052	< 0.001

	*Smooth terms*	edf	Ref.df	Chi.sq	p
	Year_s_ × Country Norway	7.009	8.078	824.3	< 0.001
	Year_s_ × Country Sweden	8.815	8.990	1753.6	< 0.001
	ID_s_	88.791	104.000	12,980.9	< 0.001

Response variable	Explanatory variables	β	SE	z	p
Proportion of calves in total harvest	Intercept	− 1.00396	0.0482	− 20.81	< 0.001
	Wolf_short_	0.409	0.112	3.65	< 0.001
	Country	0.884	0.0630	14.042	< 0.001
	Wolf_short_ × Country	− 1.324	0.131	− 10.094	< 0.001

	*Smooth terms*	edf	Ref.df	Chi.sq	p
	Lat_s_ × Country Norway	2.000	2.000	54.74	< 0.001
	Lat_s_ × Country Sweden	1.999	2.000	32.12	< 0.001
	Year_s_ × Country Norway	4.998	6.092	31.87	< 0.001
	Year_s_ × Country Sweden	7.668	8.545	1188.72	< 0.001
	ID_s_	47.029	102.000	935.66	< 0.001

Response variable	Explanatory variables	β	SE	z	p
Proportion of females in adult harvest	Intercept	− 0.260	0.0400	− 6.51	< 0.001
	Wolf_long_	− 0.0970	0.136	− 0.71	0.477
	Country	0.119	0.0595	2.00	0.0459
	Wolf_long_ × Country	− 0.383	0.170	− 2.26	0.0239

	*Smooth terms*	edf	Ref.df	Chi.sq	p
	Lat_s_ × Country Norway	3.604	4.223	18.23	0.001
	Lat_s_ × Country Sweden	6.118	6.478	20.23	0.007
	Year_s_ × Country Norway	3.505	4.351	22.53	< 0.001
	Year_s_ × Country Sweden	5.605	6.750	33.77	< 0.001
	ID_s_	11.167	102.000	28.22	< 0.001

Explanatory variables were short- and long-term wolf index (Wolf_short_ and Wolf_long_) as linear functions, and year (Year_s_) and latitude (Lat_s_) as smooth functions. Country (Norway, Sweden) was included either individually or in interaction with the other variables, with “Norway” as the reference level. The management unit ID was used as a smooth random variable (ID_s_).

### Proportion of calves in total harvest

The proportion of calves in total harvest remained close to 35% in Norway with a slight increase during the last years, but increased in Sweden from 35 to 50% during the study period (Fig. 1C). In contrast to harvest density, the proportion of calves in harvest was more strongly associated with the short-term wolf index than with the long-term wolf index (Table 1). The short-term wolf index had an opposite effect on the proportion of harvested calves in the two countries (Table 1). For Norway, the top model predicted that MMUs with average annual territory density (short-term wolf index of 0.5) were associated with a 1.23 ± 0.14 times higher proportion of calves in harvest compared to MMUs without wolves. In Sweden, the proportion of calves in harvest in MMUs with average annual territory density was 0.63 ± 0.04 times the calf proportion in MMUs without wolves (Fig. 1D). However, because the proportion of calves in harvest was initially different between MMU units in the two countries at zero or low values of the wolf index, this variable pattern resulted in similar proportion of calves in harvest at maximum wolf territory density (Fig. 1D). The proportion of calves in harvest was related to latitude and peaked at medium values (UTM N 6600000) in Sweden whereas this parameter was at its lowest at the same latitude in Norway and increased to the north (Fig. 1E).

### Proportion of females in adult harvest

The proportion of females among harvested adult moose was generally higher in Sweden than in Norway. The temporal pattern was comparable to that of the harvest density, though weaker. The proportion of females in adult harvest showed a hump-shaped form that peaked in 2010 in Norway, and two peaks around 2003 and 2012 in Sweden (Fig. 1F). The long-term wolf index was more important compared to the short-term wolf index (Table 1) and linked to a reduction in the proportion of adult females harvested in Sweden, but not in Norway (Table 2). The top model predicted that the proportion of harvested adult females in Swedish MMUs with average annual wolf territory density was 0.79 ± 0.08 times the adult female proportion in MMUs without wolves (Fig. 1G). The proportion of harvested adult females also varied with latitude, with a reduction in the northernmost parts of the study area (Table 1, Fig. 1H).

## Discussion

Harvest density of moose decreased along a spatial gradient of increasing wolf territory density in both Norway and Sweden, although to a larger extent in Sweden. In both countries, adult females were harvested less whereas the harvest of calves showed contrasting patterns between the two countries, with a decrease in Sweden and an increase in Norway with increasing wolf territory density. Our study also shows that some of the variation in harvest between the countries is related to latitude and includes different harvest responses in relation to the territory density of wolves.

Already within a 2-year perspective (where the short-term wolf index explained most of the variation) our study confirms a clear harvest response to an increase in wolf density. However, the response differed between the countries. The proportion of calves in the harvest decreased in Sweden, possibly as a functional response to the anticipated selection for calves by wolves, and increased in Norway, possibly to spare reproductive animals at a higher rate. This quick response, as well as the overcompensation found in Sweden after wolf establishment^17^ probably indicates that the realized management (harvest quotas and harvest) responds to the knowledge that wolves are present, rather than to a true reduction in moose density. With the 5-year time perspective (where the long-term wolf index explained most of the variation) both harvest density and the proportion of females in the adult harvest decreased, and was likely a result of a desired management strategy to compensate for increased moose mortality due to wolf predation. Although data on both harvest quotas and moose management plans would have been beneficial to our analyses, this information was not available for most of the MMUs included in this study. Therefore, we cannot conclude anything about the harvest-driven realization of quotas but still, our study provides novel insight of the realized harvest response on changes in wolf territory densities.

In a previous study of the effects of wolves on moose harvest in Sweden, Wikenros et al.^17^ showed a reduction in both density and composition of harvest already the first year after wolf establishment. The data and analyses in that study differ from the current study in a few important ways. First and foremost, our study includes a significantly larger dataset including both a longer period of time and a larger geographical area, which also includes adjacent parts of Norway. Second, the current dataset also included a higher wolf density in the study area, especially during the latter part of the study period. A third difference was that Wikenros et al.^17^ focused on the immediate effect of wolf territory establishment on harvest, while in this study we also included a spatial component of wolf territory density by considering individual management units. Despite these differences in study design, the results are similar, showing that the inclusion of data from two bordering countries with one common wolf population increases our understanding of how wolf territory density affects ungulate harvest.

During the study period, action was taken in both Norway and Sweden to reduce the mortality in the moose population (reduced total harvest) and to maximize productivity (reduced harvest of adult females) as a response to increased territory density of wolves. Since the production of moose calves in the population is strongly linked to the age of the adult females^29,30^, harvest of individuals with low reproductive value, i.e., those who have a low probability of producing calves next year, also promote high growth in the population^24^. In general, maximizing the number of harvested animals means that harvest should mainly be directed towards calves^16^, and this strategy also holds true in the presence of wolves^11,24^. Data from our study also confirmed a high proportion of calves in the harvest (35–50%) indicating that hunters in general traded the number of harvested animals with the amount of meat. However, whereas this strategy was more accentuated at low levels of wolf territory density in Sweden, and likely reflected a deliberate management objective to increase the proportion of calves in harvest (after year 2005, Fig. 1D), the response in this parameter to increasing wolf territory density differed between the two countries. This differential response ultimately resulted in that similar proportions of calves were harvested in MMUs in both countries at high levels of wolf territory density. In contrast, the proportion of females in the adult harvest peaked when harvest density was at its highest and were further reduced in both countries along a gradient of increasing wolf territory density, albeit at a higher rate in Sweden. Thus, although moose management in the two countries showed somewhat different strategies in changing the composition of calves in harvest as a response to the increased wolf territory density, increased wolf territory density resulted in a lower harvest in both countries.

Evaluations and analysis of the extensive dataset used in our study were partly done at a relatively course geographical scale by comparing the harvest between the two countries over a 23-year period. The fact that this data included large spatio-temporal variation in harvest was also mirrored in our analyses where year, country and latitude and the corresponding interaction terms were important predictors in combination with wolf territory density (Table 1). The spatiotemporal variation in the moose population dynamics in Scandinavia likely stems from a suite of factors including changes and variation in acceptance levels of browsing damage on commercially valuable tree species, insufficient methods for estimating local variation in forest damage levels and moose population density, and environmental variation in the landscape^31,32^. For example, variation among MMUs may stem from presence and density of other large carnivores (brown bears)^33^, immediate or delayed effects of nutritional resource competition^3^, and climatic conditions^34^.

Independent estimates on moose density was not available during the time span where harvest data was available in this study. In general, harvest data have been widely used in wildlife ecology as a proxy for population density. A study in Norway, including data from 16 moose populations, supports the use of harvest as a good proxy for population size but that harvest generally lagged behind the true population size with a few years^35^. However, in areas where large carnivores return or are present, compensation by hunters in terms of reduced harvest yield may be misinterpreted as a larger reduction in moose density than is actually the case^17^. Harvest data may therefore not be as good a proxy for ungulate density in areas with large carnivores.

In environments with little human impact, wolves may, through their predation, reduce ungulate population density and thereby indirectly reduce browsing pressure on vegetation^36^. However, in the Scandinavian forest ecosystem, characterized by strong human impact, the actual numerical impact of wolf predation on moose population growth is likely much lower. Two studies conducted in Sweden confirm a significant positive relationship between the presence of wolves and moose abundance and browsing damage on Scots pine (*Pinus sylvestris*)^37,38^. This apparently positive effect of wolves on moose abundance and browsing damage can be explained by two different, but not mutually exclusive mechanisms. First, the anticipated effect of wolves among hunters and managers on the moose population may be larger than the true numerical effect leading to an overcompensation through a reduction of harvest. This can be explained at least partly by that wolves select for moose calves which generally have a much lower reproductive value than adults, and therefore also a lower relative impact on moose population growth than expected from a regular harvest that is more proportional to moose population structure^12,30^. Second, harvest density may be lowered more than the real numerical impact of wolves due to a deliberate hunter and/or management objective to successively increase local moose population size after wolf establishment in an area. This strategy could allow for hunters to maintain an equally high future harvest yield after wolf establishment, given that the increased size of the moose population does not result in negative density dependent effects on moose population growth. However, these alternative explanations will not result in a reduction in moose browsing damage in young forest stands, which may actually be the overreaching management objective in some MMUs.

Age and sex composition of moose harvest also varied with latitude. This can at least partly be explained by differences in the physical environment, because important fitness characteristics such as reproduction and body growth in moose show large geographical variation that tend to be linked to climatic conditions and biomass productivity that vary with latitude^3,34,39^. In this area, productivity in the moose population tends to decrease towards the north, i.e., at increasing latitude^39^. However, this pattern was only partly confirmed by our response variable (proportion of calves harvested) since this variable showed the highest values at intermediate latitudes. The moose productivity gradient also suggests that the impact of wolves on the moose population will change with latitude, which has also been suggested for roe deer (*Capreolus capreolus*)^40^.

Aside from potential effects of climate (latitude), the densities of both moose and wolves are important factors that will affect the impact of wolf predation on a moose population^24,41^. The density of wolves is linked to both the number of wolves per pack and the size of wolf territories. However, because the number of wolves per pack has a relatively low impact on pack-specific kill rates^42^, the density of wolf territories is more important for the impact of wolves on the local moose population. Because the size of wolf territories also shows large variation in Scandinavia^43^, this factor, i.e., wolf territory density, constitutes an important factor of uncertainty in predicting the effect of wolf predation on the local moose population. As information about territory borders is not available from the annual monitoring of wolves in Scandinavia, the short-term and long-term wolf indices used in this study can be viewed as a method to estimate wolf territory density.

The nature of predator–prey dynamics in landscapes strongly modified by humans depends to a large extent on both direct and indirect impact by humans on all trophic levels through forestry, agriculture, harvest, and vehicle collisions^44^. For example, ungulate mortality caused by harvest is almost twice as large as the mortality caused by all terrestrial predators combined^45^. Similar to our study, harvest mortality in western North America decreased when the number of predator species increased^46^. In any system, a harvest over several years that is greater than the annual sustainable harvest will inevitably lead to reduced ungulate density and decreased harvest in the future. Our study shows that moose management in Scandinavia accounts for a growing wolf population by pro-actively respond to the increased mortality in the moose population. However, due to local and regional differences in the environment that affect moose productivity and in turn the impact of wolf predation, there is also a need for different management strategies in different areas. In general, areas with large carnivores will cause management issues to become more complex. Detailed knowledge of predation impact reduces the risk of over- or undercompensating when accounting for effects of predation on ungulate populations. There are a number of potential changes in the management system that would facilitate analyses and increase the understanding of how wild animal populations are affected by changes in the environment, including harvest and predation. Amongst these are creating conditions for linking data for management goals and harvest yield from the same management area over time. Moreover, coordination of data collection between bordering regions and between countries is also desirable and would facilitate the management of cross-border populations of wildlife.

## Materials and methods

### Study system

**Figure 2:**
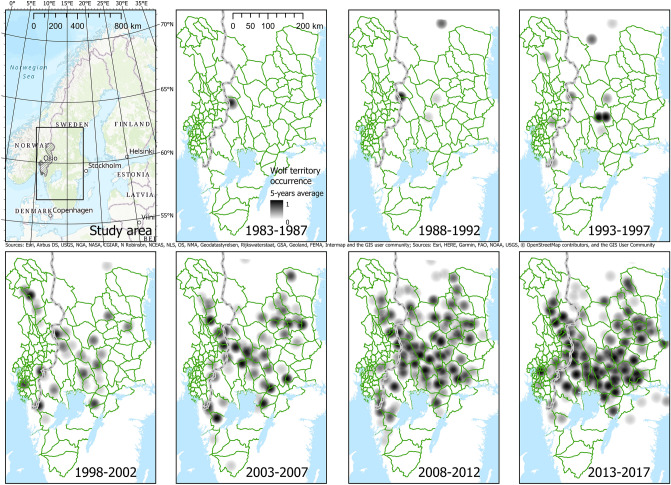
Long-term wolf index in the study area in Norway and Sweden during the period 1983–2017 (the study period in this study was 1995–2017), calculated as 5-year average annual wolf territory density by using an 18 km buffer from the annual wolf territory centre and a parabolic-shaped decaying probability of use of the area from the centre (1) to 18 km (0). The hatched area represents the Norwegian wolf reproduction zone and the green outlines the moose management units. Maps created with ArcGIS Pro 2.4.2 (Esri Inc), World Topographic Map loaded from https://cdn.arcgis.com/sharing/rest/content/items/7dc6cea0b1764a1f9af2e679f642f0f5/resources/styles/root.json.

The study was performed in 69 municipalities in four counties, covering an area of 34,986 km^2^ in South-Eastern Norway, and 27 MMUs (44,588 km^2^, average for the 23-year study period) in six counties in South-Central Sweden within and adjacent to the current wolf distribution area in the boreal vegetation zone^47^. Eleven of the municipalities were divided into two by the border of the Norwegian wolf reproduction zone. A total of 107 MMUs, 80 in Norway and 27 in Sweden, were included in the study (Fig. 2). Average area (± SE) of the Swedish MMUs (1830 ± 140 km^2^) was 3.3 times larger than the Norwegian MMUs (549 ± 86 km^2^), and the MMUs within Norway increased in size from south to north.

The climate is continental and snow covers the ground mainly during December to March. The forests are mostly composed of Norway spruce (*Picea abies*), Scots pine and some deciduous species, mostly birch (*Betula* spp.) and aspen (*Populus tremula*). Extensive silviculture has resulted in a network of gravel roads with an average density of 0.9 km/km^2^ within wolf territories^48^. Human density was 10–60 humans per km^2^ in the six counties in Sweden (https://www.scb.se/) and 8, 75, 132, and 1565 (includes the capital Oslo) humans per km^2^ in the four counties in Norway (https://www.ssb.no/) in 2017.

The wolf was declared functionally extinct in Scandinavia in 1966. In 1983, two wolves from the Finnish–Russian population reproduced in a cross-border territory between Sweden and Norway, and thereby founded the current Scandinavian population^49^. In 1991, wolves reproduced for the first time in two territories and thereafter, the wolf population started to increase in numbers and expand their breeding range in Scandinavia (Fig. 2). The monitoring 2017/18 showed that the population consisted of 72 territories of ≥ 2 wolves^50^. The current wolf population goal in Norway is 4–6 annual reproductions, while Sweden has a goal of at least 30 annual litters of pups. The wolf breeding range is restricted in both countries by legal culling, but with a smaller area allowed for wolves in Norway (i.e., the wolf reproduction zone, Fig. 2).

Moose is the main prey species of the Scandinavian wolves, with roe deer as the second most important prey^51^. Moose occur at an average winter density of 1.3 moose/ km^2^ within wolf territories^42^. Moose management is generally performed in smaller-sized administrative units in Norway compared to Sweden.

### Moose harvest data

In Norway, the municipalities are responsible for deciding moose harvest quotas and reporting number of harvested moose to a national registry. The municipalities’ objectives must be in line with the national objectives of moose management. The municipalities approve the hunting areas proposed by the hunting right holders (landowners), with harvest quotas based on a 3–5-year management plan. Several hunting areas can cooperate and make a “moose region” with a joint management plan, if approved by the municipality. Also, the municipalities are encouraged by the national management authorities to cooperate with neighbouring municipalities regarding moose management over larger geographical areas. Moose regions and management plans may therefore cover several municipalities. In such cases, one of the municipalities is given the responsibility for deciding harvest quotas, approving management plans, and reporting for all municipalities involved (“administrative municipality”). The County Governor was responsible for the moose management plans at the national scale during 1995/96–2009/10.

In Sweden, the current moose management system was introduced after a parliamentary decision in 2012 and consists of MMUs that can extend across county borders and several municipalities. Each MMU shall manage a moose population in a collaboration between landowners, hunters, stakeholders, and authorities. Each MMU prepares a 3-year management plan for its entire area that can be revised if circumstances change (e.g., establishment of wolves). The plan must be approved by the County Administrative Board, and the MMUs are responsible for coordinating census of moose density. Prior to 2012, we compiled harvest statistics (1995/96–2011/12) by linking the historically used hunting units to current MMUs for each year. We only used hunting units that could be connected to a single MMU (those that included parts of two or more MMUs were omitted) and where the area deviated a maximum of 25% from the 2017/18 hunting season.

Neither harvest quotas and moose management plans, nor data on moose density was available for the MMUs used in this study during the entire study period. Before 2012 in Sweden, calves were exposed to unlimited harvest quotas in some management units, and harvest of adults (mean ± 95% CI = 3.31 ± 0.11 per 10 km^2^) was considerably lower than the harvest quotas (4.26 ± 0.10) in management units (smaller in size than the MMUs used in this study) both in areas with and without territorial wolves^17^. In Norway, there is in many regions a spatial mismatch between the units for which harvest quotas are decided, and the units for which harvest statistics are reported. In addition, statistics on harvest quotas are not complete back in time. Therefore, we did not have access to any baseline management objectives before the return of wolves, which likely also varied between MMUs depending on the level of browsing damage on commercially valuable tree species, and moose density at the time of wolf colonization.

Due to merging of hunting areas or changes in boundaries between years’ prior 2012 in Sweden, the area with available data differs between years. Since the MMUs also varied in size in both Norway and Sweden, we compiled harvest density as the number of harvested moose per km^2^. The harvest density was calculated for all area covered by forest, bog and agricultural land. This area specification matches the definition of hunting area given by the County Administrative Boards in Sweden, but does not do so in Norway, where agricultural land is not included in the official definition. Agricultural land made up 7.3% of the Norwegian study area in 2017 (https://www.ssb.no/). For comparability, we deviated from the Norwegian definition and applied the same rule for estimating hunting area in both countries. In Norway, we used N50 map data from the Norwegian Mapping Authority to calculate the hunting area for all Norwegian MMUs. In Sweden, the hunting area came along with the data provided by the County Administrative Boards.

The harvest season in Norway starts on September 25 and lasts until the end of December, with some minor deviations for some municipalities and years. The harvest season in Sweden lasts from the second Monday in October (except in some areas in three counties where hunting was also allowed during 3 weeks in September) until the last day of January or February (two counties).

### Wolf territory density in MMUs

Wolf territory density was calculated using data from the annual wolf monitoring in Scandinavia^49,50,52^ where territorial pairs and packs are registered with minimum convex polygons (MCP) representing different territories. Estimating the size of each MCP is not a goal during the monitoring. Therefore, for each year, we calculated wolf occurrence by buffering the centre point of each wolf MCP with an 18 km radius circle, i.e., representing the average wolf territory size of 1017 km^2^^43^, and using a parabolic decay curve representing a decreasing probability of wolf territory presence from the centre point (1) to 18 km (0) (Fig. 2). This resulted in a wolf territory density raster for each year with a cell size of 1 km and cell values between 0 and 1. We extracted the average cell value for each MMU to obtain an index of wolf territory density, where 0 indicates wolf territory absence, values < 0.5 indicate that only parts of the MMU are covered by wolf territories, 0.5 indicates that the MMU is covered by averaged-size territories, and values > 0.5 indicate that the MMU contains wolf territories that are smaller than 1017 km^2^, i.e., a higher density of wolf territories.

The annual joint Swedish–Norwegian wolf monitoring occurs from October through March, during and after the moose hunting season for a given year. The establishment of new wolf territories or changes in wolf territories registered during the monitoring may therefore have occurred any time during the 1-year period from spring prior to the hunting season until the end of the winter following the hunting season. Thus, it was not possible to determine the exact time of year any change in wolf occurrence took place, and thereby the influence of wolf predation on the moose population prior to moose harvest. Therefore, we defined the short-term effect of wolves as the average wolf territory density for the previous and the current winter (short-term wolf index). For long-term effects of wolves, we used the average wolf territory density in the last 5 years, including the winter after the hunting season (i.e., range 0–4 years prior to harvest, long-term wolf index). An index of 0.5 represents an annual average wolf predation of 0.12 moose/km^2^ (95% CI 0.10–0.14), of those approximately 80% calves^41^.

### Statistical analyses

Statistical analyses were performed in R version 3.6.3^53^ using Generalized Additive Mixed Models (GAMM) in R-package itsadug^54^ version 2.4. We used a Poisson distribution with the total number of harvested moose as the response variable and the hunting area as the offset variable. For the response variables proportion of calves in total harvest, and proportion of females in adult harvest, we used logistic regression models. The ID of the MMU was used as a random variable. Because the MMUs varied in size, and small MMUs are more susceptible to random changes from year to year, we weighted the observations with the size of the MMU. As explanatory variables we included year, wolf index (short- and long-term) and latitude. Prior plotting showed that the response variables changed non-linearly over time, and w in R-package itsadug54 version 2.4e therefore entered year as smooth function. For latitude, prior plotting was inconclusive, and we therefore entered latitude as smooth term, but tried also the simplified linear term. The wolf index was entered as linear term because we expected a gradual response in moose harvest to changes in wolf territory density. We also included country (Norway, Sweden), either individually or in interaction with the other variables. We used AICc model selection to compare the performance of the short- and long-term wolf index and the latitude as linear or smooth term in the full model. Finally, we defined the best model as the model with fewest variables within ΔAICc < 4. To compare model predictions for MMUs with and without wolves, we used the R-package emmeans^55^ version 1.5.1 to estimate marginal means.

## Data Availability

The dataset analysed during the current study is available in the Dryad Digital Repository: 10.5061/dryad.zs7h44j78.

## References

[1] Hairston NG, Smith FE, Slobodkin LB (1960). Community structure, population control, and competition. Am. Nat..

[2] Messier F (1994). Ungulate population models with predation: A case study with the North American moose. Ecology.

[3] Solberg EJ, Saether B, Strand O, Loison A (2002). Dynamics of a harvested moose population in a variable environment. J. Anim. Ecol..

[4] Lavsund S, Nygrén T, Solberg E (2003). Status of moose populations and challenges to moose management in Fennoscandia. Alces.

[5] Chapron G (2014). Recovery of large carnivores in Europe’s modern human-dominated landscapes. Science.

[6] Linnell JDC (2020). The challenges and opportunities of coexisting with wild ungulates in the human-dominated landscapes of Europe’s Anthropocene. Biol. Conserv..

[7] Carter NH, Linnell JDC (2016). Co-adaptation is key to coexisting with large carnivores. Trends Ecol. Evol..

[8] Ikeda T (2019). Effects of culling intensity on diel and seasonal activity patterns of sika deer (*Cervus nippon*). Sci. Rep..

[9] Yovovich V, Allen ML, Macaulay LT, Wilmers CC (2020). Using spatial characteristics of apex carnivore communication and reproductive behaviors to predict responses to future human development. Biodivers. Conserv..

[10] van Eeden LM (2018). Carnivore conservation needs evidence-based livestock protection. PLoS Biol..

[11] Nilsen EB (2005). Moose harvesting strategies in the presence of wolves. J. Appl. Ecol..

[12] Gervasi V (2012). Predicting the potential demographic impact of predators on their prey: A comparative analysis of two carnivore-ungulate systems in Scandinavia. J. Anim. Ecol..

[13] Bassi E, Gazzola A, Bongi P, Scandura M, Apollonio M (2020). Relative impact of human harvest and wolf predation on two ungulate species in Central Italy. Ecol. Res..

[14] Gaillard J-M, Festa-Bianchet M, Yoccoz NG (1998). Population dynamics of large herbivores: Variable recruitment with constant adult survival. Trends Ecol. Evol..

[15] Caughley G (1977). Analysis of Vertebrate Populations.

[16] Sæther B-E, Engen S, Solberg EJ (2001). Optimal harvest of age-structured populations of moose Alces alces in a fluctuating environment. Wildl. Biol..

[17] Wikenros C, Sand H, Bergström R, Liberg O, Chapron G (2015). Response of moose hunters to predation following wolf return in Sweden. PLoS One.

[18] Storaas T, Gundersen H, Henriksen H, Andreassen H (2001). The economic value of moose—a review. Alces.

[19] Boman M, Mattsson L, Ericsson G, Kriström B (2011). Moose hunting values in Sweden now and two decades ago: The Swedish hunters revisited. Environ. Resour. Econ..

[20] Eurostat & Commission, E. *Agriculture, Forestry and Fishery Statistics 2013*. (Artemis Information Management, 2017).

[21] Gill RMA (1992). A review of damage by mammals in north temperate forests: 3. Impact on trees and forests. Forestry.

[22] Liberg O, Bergström R, Kindberg J, von Essen H, Apollonio M, Andersen R, Putman R (2010). Ungulates and their management in Sweden. European Ungulates and Their Management in the 21st Century.

[23] Edenius L, Ericsson G, Kempe G, Bergström R, Danell K (2011). The effects of changing land use and browsing on aspen abundance and regeneration: A 50-year perspective from Sweden. J. Appl. Ecol..

[24] Jonzén N (2013). Sharing the bounty-Adjusting harvest to predator return in the Scandinavian human-wolf-bear-moose system. Ecol. Modell..

[25] Fryxell JM, Packer C, McCann K, Solberg EJ, Saether B-E (2010). Resource management cycles and the sustainability of harvested wildlife populations. Science.

[26] Sylvén S (1995). Moose harvest strategy to maximize yield value for multiple goal management—a simulation study. Agric. Syst..

[27] Sand H, Zimmermann B, Wabakken P, Andren H, Pedersen HC (2005). Using GPS technology and GIS cluster analyses to estimate kill rates in wolf-ungulate ecosystems. Wildl. Soc. Bull..

[28] Sand H (2008). Summer kill rates and predation pattern in a wolf-moose system: Can we rely on winter estimates?. Oecologia.

[29] Sand H (1996). Life history patterns in female moose (*Alces alces*): The relationship between age, body size, fecundity and environmental conditions. Oecologia.

[30] Ericsson G, Wallin K, Ball JP, Broberg M (2001). Age-related reproductive effort and senscence in free-ranging moose, *Alces alces*. Ecology.

[31] Sandström C, Di Gasper SW, Öhman K (2013). Conflict resolution through ecosystem-based management: The case of Swedish moose management. Int. J. Commons.

[32] Dressel S, Johansson M, Ericsson G, Sandström C (2020). Perceived adaptive capacity within a multi-level governance setting: The role of bonding, bridging, and linking social capital. Environ. Sci. Policy.

[33] Tallian A (2017). Competition between apex predators? Brown bears decrease wolf kill rate on two continents. Proc. R. Soc. B Biol. Sci..

[34] Grøtan V, Sæther BE, Lillegård M, Solberg EJ, Engen S (2009). Geographical variation in the influence of density dependence and climate on the recruitment of Norwegian moose. Oecologia.

[35] Ueno M, Solberg EJ, Iijima H, Rolandsen CM, Gangsei LE (2014). Performance of hunting statistics as spatiotemporal density indices of moose (*Alces alces*) in Norway. Ecosphere.

[36] Beschta RL, Ripple WJ (2009). Large predators and trophic cascades in terrestrial ecosystems of the western United States. Biol. Conserv..

[37] Ausilio G (2018). Recolonization of Wolves in Sweden—Does it Affect Moose Browsing Damage on Scots Pine?.

[38] Gicquel M, Sand H, Månsson J, Wallgren M, Wikenros C (2020). Does recolonization of wolves affect moose browsing damage on young Scots pine?. For. Ecol. Manag..

[39] Sand H, Cederlund G, Danell K (1995). Geographical and latitudinal variation in growth patterns and adult body size of Swedish moose (*Alces alces*). Oecologia.

[40] Melis C (2009). Predation has a greater impact in less productive environments: Variation in roe deer, Capreolus capreolus, population density across Europe. Glob. Ecol. Biogeogr..

[41] Zimmermann, B. Predatory behaviour of wolves in Scandinavia. (PdD thesis, Faculty of Applied Ecology and Agricultural Sciences, Høgskolan i Hedmark, 2014).

[42] Zimmermann B (2015). Predator-dependent functional response in wolves: From food limitation to surplus killing. J. Anim. Ecol..

[43] Mattisson J (2013). Home range size variation in a recovering wolf population: Evaluating the effect of environmental, demographic, and social factors. Oecologia.

[44] Kuijper DPJ (2016). Paws without claws? Ecological effects of large carnivores in anthropogenic landscapes. Proc. R. Soc. B Biol. Sci..

[45] Darimont CT, Fox CH, Bryan HM, Reimchen TE (2015). The unique ecology of human predators. Science.

[46] Brodie J (2013). Relative influence of human harvest, carnivores, and weather on adult female elk survival across western North America. J. Appl. Ecol..

[47] Esseen P-A, Ehnström B, Ericson L, Sjöberg K (1997). Boreal forest. Ecol. Bull..

[48] Zimmermann B, Nelson L, Wabakken P, Sand H, Liberg O (2014). Behavioral responses of wolves to roads: Scale-dependent ambivalence. Behav. Ecol..

[49] Wabakken P, Sand H, Liberg O, Bjärvall A (2001). The recovery, distribution, and population dynamics of wolves on the Scandinavian peninsula, 1978–1998. Can. J. Zool..

[50] Wabakken, P., Svensson, L., Maartmann, E., Åkesson, M. & Flagstad, Ø. *Bestandsovervåking av Ulv Vinteren 2017–2018/Inventering av Varg Vintern 2017–2018*. (2018).

[51] Sand H, Eklund A, Zimmermann B, Wikenros C, Wabakken P (2016). Prey selection of Scandinavian wolves: Single large or several small?. PLoS One.

[52] Liberg O (2012). Monitoring of wolves in Scandinavia. Hystrix.

[53] R Core Team (2018). R: A Language and Environment for Statistical Computing.

[54] van Rij, J., Wieling, M., Baayen, R. & van Rijn, H. *itsadug: Interpreting Time Series and Autocorrelated Data Using GAMMs*. R package version 2.4. (2020).

[55] Lenth, R. *emmeans: Estimated Marginal Means, Aka Least-Squares Means* (2020).

