# Publisher Correction: Impact of a recolonizing, cross-border carnivore population on ungulate harvest in Scandinavia

**DOI:** 10.1038/s41598-021-89263-8

**Published:** 2021-04-29

**Authors:** Camilla Wikenros, Håkan Sand, Johan Månsson, Erling Maartmann, Ane Eriksen, Petter Wabakken, Barbara Zimmermann

**Affiliations:** 1 Grimsö Wildlife Research Station, Department of Ecology, Swedish University of Agricultural Sciences, 73091 Riddarhyttan, Sweden; 2 Faculty of Applied Ecology, Agricultural Sciences and Biotechnology, Campus Evenstad, Inland Norway University of Applied Sciences, 2480 Koppang, Norway

Correction to: *Scientific Reports* 10.1038/s41598-020-78585-8, published online 10 December 2020

This Article contains errors in the Materials and methods section under subheading ‘Statistical analyses’.

“Prior plotting showed that the response variables changed non-linearly over time, and w in R-package itsadug54 version 2.4e therefore entered year as smooth function.”

should read:

“Prior plotting showed that the response variables changed non-linearly over time, and we therefore entered year as smooth function.”

